# Menotrophin Induced Autoimmune Hepatitis

**DOI:** 10.1155/2019/7343805

**Published:** 2019-09-02

**Authors:** Shurouq H. Alqrinawi, Nuralhuda Akbar, Hind AlFaddag, Shahrazad Akbar, Lujayn Akbar, Sohail A. Butt, Mohammed Aljawad

**Affiliations:** ^1^Multiorgan Transplant Unit, King Fahd Specialist Hospital, Dammam, Saudi Arabia; ^2^Department of Medicine, Dammam Medical Complex, Dammam, Saudi Arabia; ^3^Department of Medicine, King Fahd Hospital of the University, Khobar, Saudi Arabia; ^4^Blood Bank, Dammam Regional Laboratory, Dammam, Saudi Arabia

## Abstract

Menotrophin is a protein-based hormonal therapy. It is used as a fertility medication that is given as injection either subcutaneously or intramuscularly. Menotrophin has not been previously reported to cause drug-induced liver injury. Drug-induced liver injury (DILI) is commonly seen nowadays with the expansion of the drug industry. It is associated with prescribed medications, over the counter drugs, herbal and dietary supplements. We report the first case of Menotrophin-induced autoimmune hepatitis in a 26-year-old Caucasian woman who was diagnosed with primary infertility due to failure to conceive after five years of marriage. She had received several cycles of Menotrophin, then developed new onset jaundice and fatigue associated with increase in transaminases. She had normal baseline liver function and enzymes prior to receiving treatment with Menotrophin. Evaluation showed no evidence of viral hepatitis, metabolic, alcoholic or vascular causes of liver injury. Autoimmune screening was positive for antinuclear antibody (ANA) with titer of 1 : 640 fine speckled, immunoglobulin G (IgG) level was 1900 mg/dl. Antimitochondrial antibodies (AMA) and antismooth muscle antibodies were negative. Liver biopsy showed features of chronic hepatitis with interface hepatitis and prominence of plasma cells, which best reflects autoimmune hepatitis. Her liver enzymes and bilirubin completely normalized after discontinuation of further Menotrophin therapy and starting treatment with prednisolone and Azathioprine.

## 1. Introduction

Menotrophin is a female infertility gonadotropin treatment contains follicle stimulating hormone (FSH) and luteinizing hormone (LH) purified from the urine of postmenopausal women. It binds to the follicle stimulating hormone receptor (FSH), which results in ovulation in the absence of sufficient endogenous luteinizing hormone (LH). It also binds the LH receptor, thereby stimulating proper hormone release. Menotrophin is known to cause minor gastrointestinal symptoms such as abdominal pain, nausea, vomiting, and other unspecific symptoms. No other drugs or food interaction was found.

We here present first case of drug-induced autoimmune hepatitis (DIAIH) following treatment with Menotrophin requiring immunosuppressive therapy that has hitherto not been described to best of our knowledge.

The causality of autoimmune hepatitis (AIH) is uncertain, but the disease can be triggered in some patients by external factors such as viruses or drugs. DIAIH can mimic many liver diseases, and its effect can range from a mild elevation of liver enzymes to liver failure.

Many drugs have been linked to cause AIH, which sometimes persist after drug discontinuation, suggesting that they awaken latent autoimmunity. Drug-Induced Autoimmune hepatitis (DIAIH) is still a poorly defined and an under-reported liver disorder. Histologically distinguishing DILI from AIH remains a challenge.

## 2. Case Presentation

A 26-year-old Caucasian lady was diagnosed with primary infertility after failure to conceive despite ongoing attempts over five years. The patient received four cycles of treatment with Menotrophin within the last four years with approximate interval of 12 months in between cycles. Each cycle consisted of five doses of Menotrophin given as intramuscular injection every other day. One month after the last cycle, the patient started to develop yellowish discoloration of sclera and skin associated with pruritus, which was gradual and progressive with time. She also developed pale stool, and dark urine. There was no history of vomiting, nausea, abdominal pain, change in bowel habit, or weight loss. She had No history of alcohol consumption, and denied using recreational drug, other prescription or nonprescription drugs or herbal supplement. The patient is not known to have any prior medical conditions and no known autoimmune disorders.

On examination, vital signs were within normal limits. Despite being deeply jaundiced, she had normal level of consciousness and had no stigmata of chronic liver disease and negative abdominal finding. She had baseline investigations prior to starting Menotrophin that showed normal liver enzymes and liver synthetic functions. Her laboratory tests during this presentation showed Aspartate aminotransferase 590 U/L, Alanine aminotransferase 504 U/L, Total Bilirubin17.81 mg/dl, Gamma-glutamyl transferase 486 U/L, Direct Bilirubin 13.16 mg/dl, albumin 2.4 g/dl, alkaline phosphatase 366 U/L, International normalized ratio 1.3, Hemoglobin 11 g/dl, total white blood cells 9.25 × 10^9^/L, platelets count 188 × 10^9^/L. Further workup revealed immunoglobulin G (IgG) level 1900, B2 glycoprotein (IgG) 42.4 EU/ml, ANA 1 : 640 fine speckled.

AMA, antismooth muscle antibodies (ASMA), B2 glycoprotein (IgM), proteinase 3 antibodies (PR3), liver Kidney Microsomal antibodies (LKM), hepatitis B and C as well as EBV and CMV serology were all negative.

Liver ultrasonography showed coarse echotexture with lobulated surface and enlarged caudate lobe, no portal vein thrombosis, normal biliary, no stones. Unfortunately, there is no previous baseline liver ultrasound for comparison.

**Figure 1:**
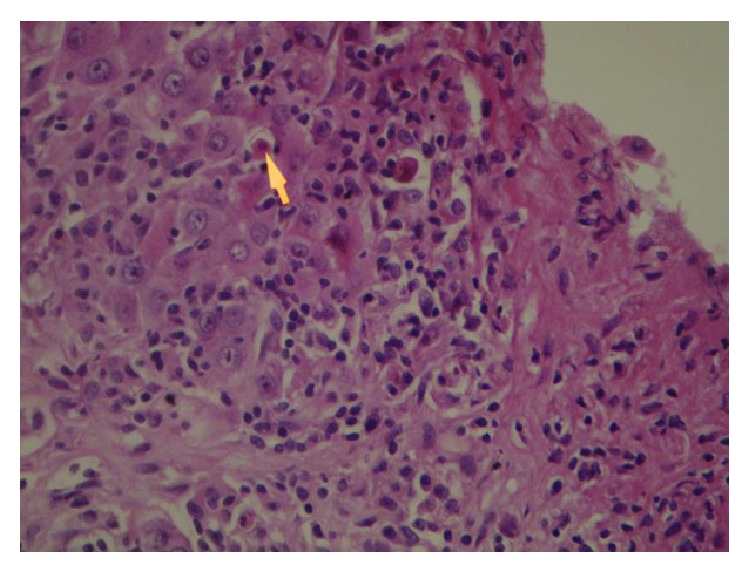
Liver biopsy showing Interface hepatitis revealing spillover of inflammation into adjacent liver parenchyma and necrotic hepatocytes. HE 10 × 20.

**Figure 2:**
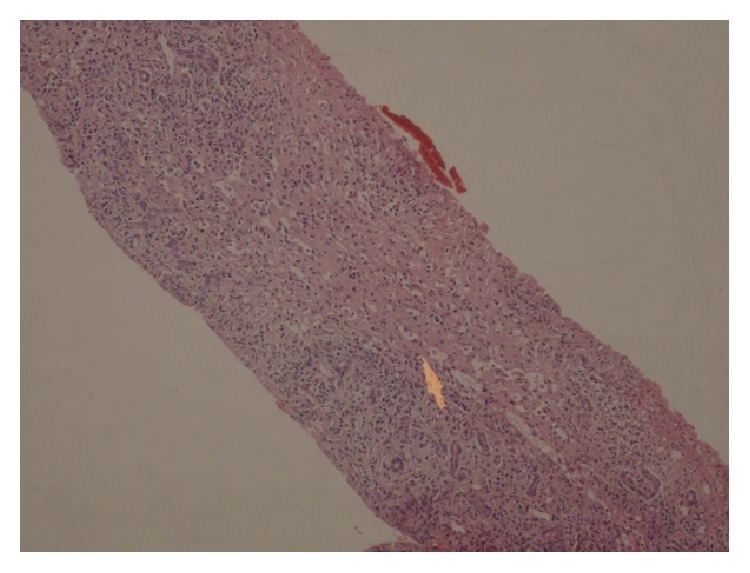
Liver biopsy showing portal expansion and bridging fibrosis. HE 10 × 10.

**Figure 3:**
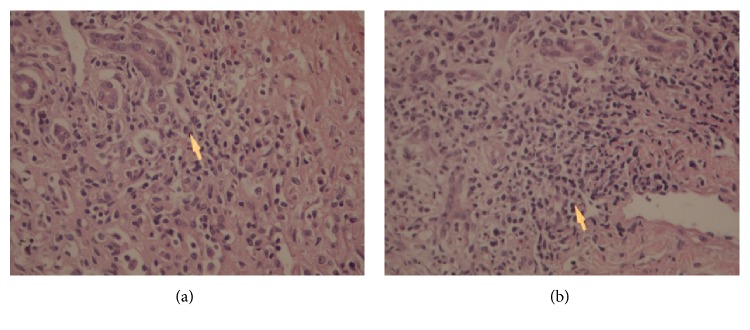
Liver biopsy showing moderate to marked portal tract mostly lymphoplasmacytic inflammatory cell infiltrate. HE: 10 × 20.

Liver biopsy revealed portal fibrosis with expansion and formation of portal-portal and portal-central bridging fibrosis. Marked mostly lymphocytic infiltrate containing plasma cells and occasional polymorphs & esosinophils were seen in portal tract, extending focally into the lobules with moderate interface & lobular hepatitis. No obvious hepatocyte rosette formation, though a few necrotic hepatocytes were present. The bile duct revealed focal injury through infiltration by polymorphs and lymphoid cells. The features of liver biopsy suggested chronic active hepatitis with interface hepatitis and prominence of plasma cells stage 3 (Figures 1, 2, 3(a) and 3(b)).

The diagnosis of autoimmune hepatitis was based on Simplified diagnostic criteria of International Autoimmune hepatitis Group with a score of 7 points, which make her case fall in the category of definite autoimmune hepatitis [1]. After establishing the diagnosis of AIH, she was started on prednisolone and Azathioprine. Her liver enzymes and synthetic function improved with time and then normalized after 4  weeks of treatment (Table 1). Her prednisolone was tapered down until complete discontinuation and maintained on Azathioprine monotherapy.

## 3. Discussion

Infertility has psychologic, emotional, and financial consequences on families. The prevalence of primary infertility in the United States in married women is between 15 and 44 years is 6% [2]. However, with medical development, advanced fertility treatment options are more numerous and variable than ever.

Menotrophin is a female infertility gonadotropin treatment which may induce minor gastrointestinal adverse effects such as abdominal pain, nausea, vomiting, and unspecific symptoms.

DILI is a common cause of liver disease that could be associated with prescribed medications, over the counter drugs, herbal and/or dietary supplements. Its manifestations can mimic many liver diseases, and its effect can range from a mild elevation of liver enzymes to liver failure. DILI accounts for 10%–50% of elevated liver enzymes in adults, and 10% of all acute hepatitis cases [3]. Risk factors include (a) age; adults are at higher risk than children except for that of valproic acid-induced DILI, which is more common in children; (b) women are at higher risk than men; (c) alcohol abuse; (d) malnutrition [4]. Few DILI cases may present with typical features of autoimmune hepatitis, which makes differentiating between the two challenging. DIAIH is still a poorly defined and an under-reported liver disorder. It can commonly be caused by minocycline, alpha-methyldopa, nitrofurantoin, hydralazine, and statins [5].

Diagnosis of AIH could be challenging and therefore several scoring systems have been developed to standardize the diagnosis. The Simplified diagnostic criteria of International Autoimmune hepatitis Group Score showed 81% sensitivity and 99% specificity when using a cutoff score ≥7 points as a definitive diagnosis of AIH [1].

In this case we present here, our patient scored 7 points; one point for positive ANA, two points for positive IgG, two points for liver histology & two points for absence of viral hepatitis, which qualify as definitive diagnosis of AIH.

DILI is still a diagnosis of exclusion especially with the lack of gold standard test or tool to establish the causality. The RUCAM (Roussel Uclaf Causality Assessment Method) or its previous synonym CIOMS (Council for International Organizations of Medical Sciences) score is a tool that could provide help in guiding a systematic and objective evaluation of patients suspected to have DILI [6,7]. RUCAM score ranges from −9 to +10 with higher score indicating higher likelihood of DILI [6]. The diagnosis of DILI in our patient is classified as probable based on RUCAM score of +6.

It is important to distinguish drugs as triggers of a self-perpetuating autoimmune liver disease from immune-mediated drug-induced liver injury (IM-DILI). Immune-mediated DILI nearly always resolves or becomes quiescent when drugs are withdrawn. Another possibility is that AIH was quiescent and remains undiagnosed until a drug triggered a new autoimmune process. Thus, to attempt to make a proper diagnosis of the type of immune process affecting the liver is challenging [5]. The presence of liver cirrhosis was surprising in this young patient which could suggest the possibility of unrevealed chronic liver disease with Menotrophin acting as a trigger to the recent inflammatory activity.

In addition, recognition and discontinuation of the drug causing DIAIH is the most crucial step in management, along with evaluating the severity and the characteristics of injury. Liver biopsy is not essential for diagnosis. The primary treatment is with corticosteroid therapy.

Clinical pictures and histopathological features of DILI and AIH are similar, but the key point of differentiation between the two is the response after cessation of immunosuppressive therapy. Successful cessation has been reported in DIAIH, while relapses has been reported in AIH [8].

## 4. Conclusion

This report describes a case of probable drug-induced autoimmune hepatitis following treatment with Menotrophin for infertility in a young female with no previous known liver disease and with documented normal liver enzymes prior to therapy. We suggest checking liver function test prior to staring and during such treatment, since there is a probability of serious DILI associated with its use.

## Figures and Tables

**Table 1:** Liver enzymes improved with the treatment.

	AST	ALT	ALP	Total bilirubin
Reference range	0–37 U/L	0–45 U/L	46–116 U/L	4–21 mmol/L
1^st^ day of treatment	593	482	343	57.3
5^th^ day of treatment	278	286	318	47.47
15^th^ day of treatment	62	103	226	30.6
21^st^day of treatment	36.3	45	176	22.1
28^th^ day of treatment	31	39	122	20.1
